# The EEG-Based Attention Analysis in Multimedia m-Learning

**DOI:** 10.1155/2020/4837291

**Published:** 2020-06-10

**Authors:** Dan Ni, Shuo Wang, Guocheng Liu

**Affiliations:** The School of Information Engineering, Jilin Engineering Normal University, Changchun, 130000 Jilin, China

## Abstract

In recent years, research on brain-computer interfaces has been increasing in the field of education, and mobile learning has become a very important way of learning. In this study, EEG experiment of a group of iPad-based mobile learners was conducted through algorithm optimization on the TGAM chip. Under the three learning media (text, text + graphic, and video), the researchers analyzed the difference in learners' attention. The study found no significant difference in attention in different media, but learners using text media had the highest attention value. Later, the researchers studied the attention of learners with different learning styles and found that active and reflective learners' attention exhibited significant differences when using video media to learn.

## 1. Introduction

The industrial revolution 4.0, also known as the digital revolution, has driven a massive transformation in the world [1]. Currently, the use of digital technology has occurred in all aspects of life and various age groups. People have been accustomed to accessing various knowledge from the Internet.

The rapid development in digital technology has also created new challenges to conventional education systems at various levels of education, from basic education to higher education [2]. The popularity of mobile terminals and the rapid development of the mobile Internet have provided a carrier for the development of mobile learning (m-learning) and have made mobile learning a hot research topic in education informatization [3]. The size and weight of tablets, as well as improved screen quality, have made reading from mobile devices more acceptable [4]. Mobile learning has attracted much attention as a new teaching and learning model. Mobile learning (m-learning) has become increasingly popular in universities, and more college students have access to smartphones, tablets, and other mobile devices [5]. Digital technology has also changed students' tendency to learn. Today, students tended to demand the freedom to decide what they want to learn and when and how they want to learn [1]. A common factor in mobile learning was the learning media, and people had an interest in further analysis of the selection of appropriate media for educational content to achieve higher learning results [6]. There was a wide range of perspectives on the use of information technology for achieving learning effectiveness, ranging from those who asserted that media did not influence learning effectiveness to those who believed that the decisions made regarding media would influence learning effectiveness [7].

Today, common types of contents were accessed via mobile devices, including videos and texts such as pdf, audio, or video files or a combination of these file types evidenced in e-books and online articles. Different kinds of content had the potential to support learning through both verbal and visual demonstrations that might motivate students to learn [7]. A study showed that there might be an interaction between media choice and other variables, which not only affected perceived usefulness but also affected the learning effect of educational programs [8].

Most research on mobile learning focused on learner motivation and attitudes [9]. Hwang and Wu [10] pointed out that the limitations of mobile devices also affected students' learning attention and cognitive load. Solving the problem of attention was a topic worth exploring in mobile learning, but the research on this problem was insufficient [11].

After entering the 21st century, the increasingly mature brain imaging technology has once again promoted cognitive science to move forward. Using neuroscience research methods and techniques to study classical cognitive problems has become a new trend in academia. Therefore, people's research on cognition has also achieved a breakthrough from macrobehavior to the microneural connection [12]; EEG was one of the research tools used to measure human brain activity. Students' learning involves brain activities of information input and processing because the use of EEG to measure students' learning status is a good choice.

Therefore, this study focused on the differences in learners' attention when using three different learning media for mobile learning based on EEG.

The purpose of the research is to use EEG technology to measure mobile learners' attention differences in different learning media environments and then to clarify the impact of different media on mobile learners' attention, to provide corresponding suggestions for learners, teachers, and e-learning resource developers, which were related to the trend of improving students' mobile learning, to support individuals to better improve mobile learning performance. Researchers were motivated to design and research a mobile learning experience with different media learning materials, which would be supported by tablet devices. In this process, we took learning style theory as a theoretical perspective to compare the differences of the learners' attention.

The research questions are as follows:

1. When learners used three media for mobile learning, which case has the highest learner attention value?
2. When learners used three media for mobile learning, would gender, education affect learners' attention?
3. When learners use three media for mobile learning, was there a difference in attention among students with different learning styles?

## 2. Literature Review

### 2.1. Mobile Learning

Mobile learning has currently no more unified concept. Researchers had different definitions of mobile learning from different perspectives. From a technical point of view, mobile learning was a kind of learning that could be done by learners at any time and any place with the help of mobile computing devices that could effectively present learning content and provide two-way communication between teachers and students [13]. From the learner's point of view, mobile learning was that learners used mobile devices to learn anywhere and anytime [14]. Researchers of China believed that mobile learning referred to a new form of learning that used wireless mobile communication network technology and wireless mobile communication devices to obtain educational information and resources and educational services [15]. Definitions of mobile learning emphasised mobility, access [16], immediacy [17], ubiquity [18], and convenience [17].

In summary, mobile learning covered the following points: any place, any time, any form, mobile network, and equipment support. In short, mobile learning referred to a kind of learning model that could achieve access to digital learning resources and educational information anytime and anywhere, with the help of the seamless wireless network, and portable mobile communication equipment, and facilitate communication and interaction.

#### 2.1.1. Attention in Mobile Learning

In e-learning and mobile learning, lack of student attention has become an important problem [11], and some scholars have confirmed that attention was closely related to academic performance [19]. Although e-learning or mobile learning had the advantage of not being limited by time and place, there were also related issues caused by the non-face-to-face learning environment of teachers and students, especially the screen size of mobile learning devices and the characteristics of their representation methods [20]. Attention was still an urgent problem. Assessing students' attention status in an e-learning environment was more difficult than assessing them in face-to-face instruction [21]. With the advancement in the evaluation of human physiological signals, e-learning research increasingly used physiological signals to determine students' level of attention [19]. In recent years, some researchers have started using EEG detection tools [22, 23] for empirical research on student attention and attention during multimedia learning [24].

#### 2.1.2. The Learning Style in Mobile Learning

There were many different definitions of learning styles, among which were more influential: Kinsella [25] proposed that learning style referred to the partial way in which learners were naturally employed in the process of information and information processing.

Studies have shown that learning styles and learning environments had a positive effect on learners' academic performance [26]. Learning style was also a factor that affects the quality of e-learning [27] and greatly affected learners' academic performance [28]. Learning styles also affected mobile learning [11].

The most widely used in mobile learning research was the learning style scale developed by Felder and Solomon (1991), which was divided into four dimensions (information input, information processing, content understanding, and perception) (see Table 1). Learners had different characteristics in each dimension, and each dimension had two attributes (visual/verbal, sensing/intuitive, active/reflective, and sequential/global). ILS was compared with other learning style questionnaires; the results showed that this questionnaire was more comprehensive, and the results are better [29]. This scale was widely used in e-learning research.

The learning style was added in this study to confirm whether students with different learning styles had different attention when using different media for mobile learning.

### 2.2. Learning Media

The influence of the media on learning has been a research topic that researchers pay attention to. Liu et al. [31] studied the impact of different media on e-learning content, including the impact of text and video on users' acceptance of e-learning and user attention. They found that the richness of content was positively correlated with user focus.

According to cognitive theory, learning was the acquisition or reorganization of the cognitive structures through which humans processed and stored information [32]. It focused on information and content delivery in mobile learning using multimedia learning (dual code, cognitive load theory): images, audio, video, text, and animations [33].

The main focus of media debate was whether one media naturally promotes learning more effectively than the other [34–36]. The answer to this question was mainly divided into two opposing views represented by Clark and Kozma. Clark [37] believed that the type of media did not affect learning; learning was only affected by the way the media was used. This view suggested that video-based learning materials were not necessarily more effective than text, because how text-based or audio-based applications were designed should promote equal levels of learning [38]. In contrast, Kozma [39] believed that different types of media “had special characteristics, which made them more or less suitable for some types of learning tasks.” A set of computer-based experiments were carried out, which used three different media combinations (text only, text and diagrams, spoken text, and diagrams) to study learners' understanding achieved in complex areas (statistics) in a real e-learning environment [30].

This research was to provide the learning content of three media in a mobile learning environment and analyze the differences in learners' attention.

### 2.3. Attention and EEG

EEG was a process used to record brain wave activity and was called the “mind window” [40]. In human-computer interface research, EEG was a method for making psychophysiological measurements to check the relationship between psychological processes and physical processes. In general, EEG was measured by recording the voltage of the electrodes on the scalp. The electrodes were placed at designated positions allocated on the head [41]. In recent years, EEG technology has also been widely used in other fields, especially in computer interface design and computer game development. Brain-computer interface research focused on using EEG activities to control external devices, such as robots and virtual environments [42].

Currently, as a research tool, more and more researchers used portable EEG for educational research and have achieved many research results [43–45]. To a certain extent, investment in attention could also promote brain information processing and coding so that learners could obtain better academic performance [46]. Since it was difficult to measure attention using self-reporting tools, many studies have used electroencephalography (EEG) as a tool to measure changes in attention status [47]. Attention was the most commonly used evaluation index of portable EEG technology in education research [48]. If we could use EEG technology scientifically, it could be an effective tool for detecting and processing brain signals for educational purposes. More precisely, when using EEG technology in some innovative ways, it could capture brain signals and process them to determine learners' learning and memory during learning [49]. Studies identified that the EEG data was used successfully in detecting the learners learning style and learning preferences and the correlation between them [50].

## 3. Research Methodology

### 3.1. Participants

In this study, 30 college students from a university of science and technology in China were randomly recruited by snowball sampling, all of whom were right-handed. Because the test data of two participants were unreliable, they were ignored. Among the 28 participants, 19 were male, 9 were female, and the age was between 19 and 31 years old (*M*_age_ = 24, SD = 2.78). Among them, 13 were undergraduates and 15 were postgraduates. According to the investigation of the participants, they had no mental diseases such as epilepsy, depression, and hyperactivity disorder or did take psychoactive drugs for a long time. At present, they neither had used any drugs to change their thinking nor had any history of head injury or brain injury. The experimenter introduced the scope and procedure of the experiment to the participants and informed them that the experiment would not cause any risk to their health, to ensure that the participants could participate in the experiment voluntarily and sign the informed consent before the experiment. All participants had more than 5 years of mobile device experience, and 27 people had more than 2 years of experience in using tablet computers, which showed that they are familiar with the operation of mobile devices.

Because this experiment was based on the learning of mobile devices, the participants needed to ensure that they can see the learning content, so they were required to wear appropriate glasses when necessary to ensure that the learning content is clear and visible, and the test materials were in Chinese, that is, the native language of the participants. The background characteristics of the participants are shown in Table 2.

### 3.2. Stimulus Materials

To ensure that the experimental content can be scientific and reasonable, the researcher invited two experts with rich experience in mobile learning and EEG. After repeated discussion, research, and trial, the final experimental materials were determined to choose the Xinhua News Agency website, the most influential online media in China and the Chinese website with global influence (http://www.xinhuanet.com) and determined three declarative knowledge with similar themes, and the same difficulty was presented in three different media forms: text, text + graph, and video. This was a combination of multimedia demonstrations used in many e-learning environments. Based on determining the content, the researcher arranged five college students to take a test. After feedback, the content was similar and the difficulty was the same, and the required learning time was close. The media types and contents selected in this experiment are shown in Table 3.

### 3.3. Research Instruments

#### 3.3.1. EEG Device and Algorithm Optimization

In this study, the MindWave mobile headset produced by the NeuroSky company was selected as a tool to record EEG data signals. This equipment had a great influence on students' attention training. Previous studies [19] have confirmed that NeuroSky's mindset headset was effective and reliable enough based on the correlation between bird watching scores. Also, Rebolledo-Mendez et al. [51] also found that NeuroSky's mindset headphones measured a positive correlation between attention measures and self-reported attention levels through the second life assessment exercise. The results showed that the attention value measured by NeuroSky's mind headset had satisfactory validity and reliability for recognizing the attention of learners in learning activities. The new generation of the MindWave mobile headset was more perfect based on a mindset headset. The device ran well, had good measurement accuracy, and had its software development kit (SDK), which was convenient for software developers to design applications [52].

**Figure 1:**
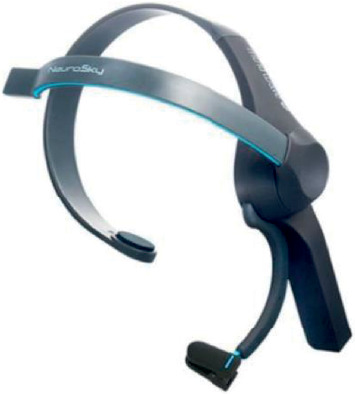
MindWave mobile headset.

The MindWave mobile headset was composed of an ear clip and a sensor arm (see Figure 1). The reference electrode and the ground electrode of the earphone were located on the ear clamp, and the EEG electrode was located on the sensor arm and the left forehead above the eyes. The EEG signal of the forehead could be detected in real time. These EEG signals could show the changes in people's consciousness after the complex e-sense TM calculation. By quantifying the psychological state of the subjects as the value of attention, which could be used to analyze the degree of attention of learners, it could be divided into attention and relaxation, and the parameters were between 0 and 100. The device also had noise-filtering technology, through a complex algorithm to eliminate interference from other electronic equipment or daily living environment.

The MindWave mobile headset used the TGAM module to process and output brain wave spectrum, EEG signal quality, original brain wave, and two NeuroSky e-sense parameters: attention and relaxation detection. The NeuroSky system was composed of dry electrodes. The interface with the human body only needed a simple dry contact. A single EEG channel had three contact points: EEG (EEG acquisition point), ref (reference point), and GND (ground point). The original EEG data was output at 512 Hz.

**Figure 2:**
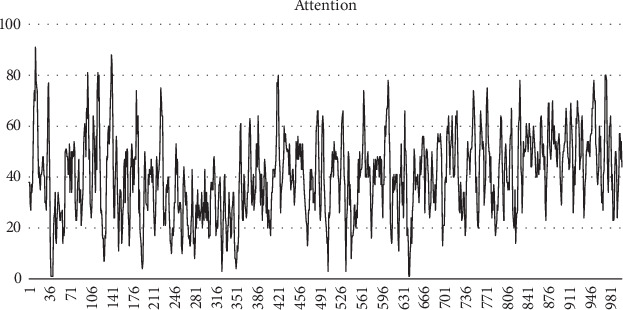
Attention data calculated by the chip's algorithm.

As the attention data calculated by the chip's algorithm still needed to be improved in the application, such as large fluctuations and delay (see Figure 2), at the same time, the data was divided into many meaningless null regions. This increases analysis cost. To ensure that scientific and accurate data was collected in this experiment, the researchers optimized the algorithm. The main methods included the following: (1) a checksum check was added, that is, checking whether the data value of the data packet and the checksum data used for detection are consistent, (2) cross-checking with the previous data packet was performed, and (3) the attention was stepped and the average value of the two continuously updated attention values was used to make the jittered data smoother.

#### 3.3.2. Index of the Felder-Solomon Learning Style (ILS)

The ILS questionnaire was composed of forty-four items requiring students to choose from two options, a or b, compulsorily. Each number was referred to any of the four scopes or dimensions which included the following: active and reflective, sensing and intuitive, visual and verbal, and sequential and global. For the scoring, summing up the number of *a* and *b* responses for each dimension formed scores which range from 1 to 11. Lower scores were subtracted from the higher score of either a or b.

#### 3.3.3. The Participant Questionnaire

Participants' questionnaire collected demographic information about participants' gender, education background, age, participants' experience of using tablet computers and mobile devices, and learning time spent on mobile devices every day. At the same time, participants also had to answer the content test.

### 3.4. Experiment Design and Procedure

#### 3.4.1. Experiment Design

Participants in the experiment read three different media types of learning content on the tablet computer, and each of them freely chose the reading order according to their wishes. Each reading content was about 5 minutes, and after the experiment, they filled in the test questions.

#### 3.4.2. Experiment Procedure

The experiment was carried out in a very quiet research room. The curtain was put down to avoid direct sunlight, and the indoor light source was turned on. After confirming that there was no problem with the tablet computer, MindWave mobile headset, and data storage terminal (computer), two research assistants explained and demonstrated the experiment. The participants completed the following tasks.

**Figure 3:**
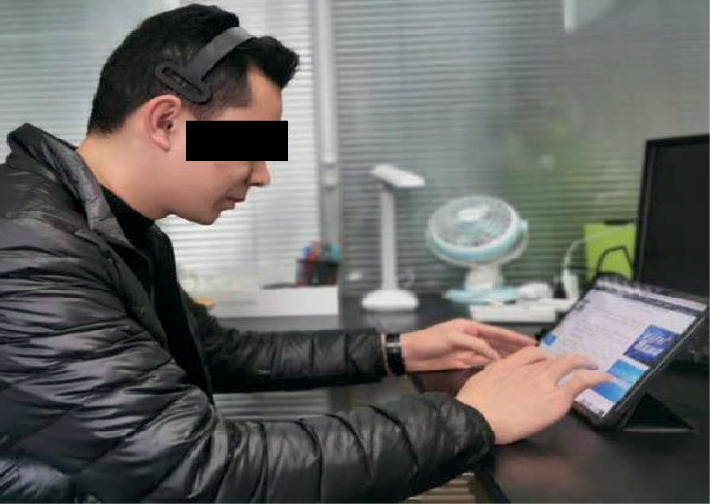
EEG measurement process. The participant was being tested while using an iPad.

Each participant signed an informed consent form and found a suitable position to sit down, facing the tablet, correctly wore the EEG headset with the help of the researchers, and confirmed that the device was correctly placed, and the participants were told not to move as much as possible. The participants closed their eyes in a relaxed way (starting from the stable EEG data), opened their eyes, and began to read at their own pace after a few seconds. After each reading, they should answer the corresponding test questions. During this period, they were not allowed to return to the article to find the answers. When one content test was over, the participants closed their eyes and had a rest for about 15 seconds, then moved to the next one, and so on, until the three content tests were over (see Figure 3).

After the test, each participant would receive a survey about learning style and demographic information, followed by a reading behavior survey, and then, the researchers reported the results to each participant and thanked them for their participation. The whole process lasted 30-40 minutes, depending on how fast participants read each paragraph.

### 3.5. Data Collection and Analysis

According to the calculation method of the learning style scale, the researcher made statistics on the collected learning style questionnaire. After putting each answer in the corresponding question, the researcher can finally sum up the category of student learning style, which is represented by number + a/b. Each calculation result was finally recorded in an Excel spreadsheet.

The data collected by EEG is directly transmitted to the computer through Bluetooth and saved in Excel. Based on the relevant previous research, the average value of the participants' attention in the data information was selected for analysis and research.

The independent sample *t*-test in SPSS 25.0 statistical software was mainly used for data analysis.

## 4. Experimental Results

In this section, we analyzed the attention from the EEG experiment and answered the following questions:

1. Were there differences in the learner's attention in the context of three learning media (text, text + graphic, and video)?
2. Did the gender and education affect learner attention on learning media?
3. Did learning styles affect learners' attention differences in different media?

### 4.1. Difference Comparison of Attention Values of Learners in the Three Media Contexts

From the results, we could get that the average attention value of the text media (*M* = 55.43, SD =12.82) was higher than others, including text + graph (*M* = 51.32, SD =10.83) and video (*M* = 51.18, SD =11.55) (see Table 4). It could be seen that there was no significant difference in the attention of media form when using text + graph or video, and the attention of text media showed a higher mean, which could prove that text media had a certain promotion effect on the attention of the learners.

### 4.2. Difference Comparison of Attention Values of Learners on Gender

Through the independent sample *t*-test, there was no significant difference of attention on gender when using text (*t* = −0.941, *p* = 0.356), text + graph (*t* = 0.475, *p* = 0.639), and video (*t* = 1.261, *p* = 0.219), but we found that both male and female learners have a higher attention value on text media than the other two media. Meanwhile, the average male attention value was significantly higher than the female's (see Table 5).

### 4.3. Difference Analysis of Attention Values of Learners with Different Education Experience

As shown in Table 6, there was no significant difference of learners' attention with different education experience when using text (*t* = −1.532, *p* = 0.138), text + graph (*t* = −1.472, *p* = 0.153), and video (*t* = −1.746, *p* = 0.093). Graduates and undergraduates had a higher attention value (*M* = 58.80, 51.54) when using text media to learn than text + graph (*M* = 54.07, 48.15) or video (*M* = 54.60, 47.23). Moreover, the average attention value of graduate students was higher than undergraduates'.

### 4.4. Difference Comparison of Attention Values of Learners with Different Learning Styles

The first axis of Felder's learning style is active and reflective, so students are divided into two categories based on the survey results. As shown in Table 7, through the independent sample *t*-test, the results showed that the attention difference was not significant between the active learners and reflective learners when using text or text + graph. However, there was a significant difference in attention between active and reflective learners when using video media (*t* = −2.308, *p* < 0.05); it indicated that when using video media to learn in a mobile learning context, the attention of active learners (*M* = 46.15, SD = 9.23) significantly was lower than that of reflective learners (*M* = 55.53, SD = 11.86).

Since almost all the research subjects belong to the perception type and the visual type, no attention difference analysis was conducted.

In the group of sequential and global learning styles, the data (see Table 8) showed that whether in text (*t* = 0.203, *p* = 0.841), text + graph (*t* = 0.851, *p* = 0.403), or video (*t* = 0.598, *p* = 0.555), there was no significant difference in learner attention between sequential and global learning styles.

## 5. Conclusions and Discussion

### 5.1. Research Conclusions and Contributions

This paper has made an empirical study on the attention of learners on different learning media in a mobile learning environment through questionnaires and psychophysiological methods (EEG recording). This study improved the stability and accuracy of the attention data output by the TGAM chip without increasing the real-time data transmission delay, optimized the impact of blinking on EEG data, and improved the expressiveness of the data. The researchers have chosen 28 college students as the participants and the NeuroSky MindWave headset as the tool to measure attention based on EEG. Through the EEG experiment, we have analyzed the attention difference of students on three media forms (text, text + graph, and video) and have investigated the influence of gender, education, iPad experience, and learning style factors on a student's attention.

The learners expressed greater attention in text media, which possibly resulted from the fact that text media had only words that were easy to concentrate; however, the difference did not reach statistical significance. Also, the male's attention was greater in the three media than the female's, graduate students' attention was greater than that of undergraduates, and participants who used an iPad for more than five years showed stable attention.

According to learning styles, there was a significant difference in attention on video between active learners and reflective learners though there was no significant difference between sequential and global learners. The reason for the results might be related to the nature of the experimental material, which might require further research by using more sensitive learning style tests.

According to this study, in mobile learning, teachers or instructional designers could provide more text media materials for college students, rather than other multimedia. Teachers or instructional designers should appropriately improve the attention of female college students in mobile learning. Experiments have also shown that graduate students' attention was much greater than that of undergraduates. Therefore, appropriate design or strategies would be used to improve the undergraduate's learning attention in mobile learning. In mobile learning, according to the results of learning style measurement, teachers or instructional designers should provide appropriate learning media for students; in particular, reflective learners were more suitable for learning with video media than active learners.

The results of this study confirmed the relationship between media and attention using traditional questionnaires and psychophysiological measures. The results also showed that psychophysiological measures could be used to study learning behavior.

The study had some contributions to related literature. First, the study confirmed that learners' attention is different when using different media in a mobile learning environment, especially video media. Although previous studies have investigated this relationship, they have not focused on mobile learning environments.

Another contribution of this study was the use of EEG recording for research. In the existing studies, it was not sufficient to measure the impact of media on learners in mobile learning only by a questionnaire. This study suggested the use of EEG recordings to research mobile learning, rather than the traditional paper-and-pencil questionnaire.

This study provided a way to study the effect of learning attention. It was necessary for educators or developers of e-learning systems to design appropriate media learning materials based on learners' learning styles.

### 5.2. Limitations and Suggestions for Future Research

The research has certain limitations but also provides some suggestions for future research. First of all, three types of learning media were regarded as the important premise of affecting learners' attention in a mobile learning environment. However, many factors might be considered in future research.

Secondly, compared with the objective tools of EEG, the method of the learning style questionnaire was subjective; thus, this study is not completely objective.

Thirdly, this study used a NeuroSky MindWave headset to measure the attention. Future research should try to analyze the original EEG data (alpha, beta, gamma, delta, and theta), which could lead to more accurate experimental data.

Fourth, experiments were complex and easily affected by the surrounding environment; in order to ensure the cycle of the experiment, the sample size was small, which was a limitation of this study. As the sample size was very limited, the results of statistical analysis were not easy to explain. In EEG studies, the sample size was usually small [42]. In future studies, we would try to increase the sample size and confirm whether the results are valid.

Fifth, the data used in this study was the average value of attention. In most EEG experiments, the average value of attention was selected. In future research, we would try to follow the value of attention at specific time research to explore more effective research methods.

## Figures and Tables

**Table 1:** Brief description of the types of ILS.

One polarity	Opposite polarity
(i) Active learners prefer rushing in and doing	(i) Reflective learners prefer to reflect before starting
(ii) Sensing learners prefer facts and prefer using well-known relationships	(ii) Intuitive learners prefer to discover possibilities and relationships
(iii) Visual learners prefer pictures and visual material	(iii) Verbal learners prefer written and spoken text
(iv) Sequential learners tend to learn material in steps	(iv) Global learners absorb material often randomly without necessarily seeing the connections

This table was drawn from [30].

**Table 2:** Background characteristics of participants.

	*N*	Percentage (%)
Gender		
Male	19	67.9
Female	9	32.1
Educational background		
Undergraduate	13	46.4
Graduate	15	53.6
Age		
Under 20	2	7.1
21-25	20	71.4
Above 26	6	21.4
Tablet experience		
Less than 1 year	1	3.6
2-4 years	15	53.6
Above 5 years	12	42.9

**Table 3:** Experimental material design.

Media forms	Contents	Estimated learning time
Text	Artificial intelligence	5 min
Text + graph	Virtual reality	5 min
Video	5G technology	5 min

**Table 4:** Learners' attention value in the three media contexts.

	*N*	*M*	SD
Attention (text)	28	55.43	12.816
Attention (text + graph)	28	51.32	10.829
Attention (video)	28	51.18	11.554

**Table 5:** Difference comparison of attention values of learners on gender.

	Gender	*N*	Mean	SD	*t*	*p*
Attention (text)	Male	19	57.00	14.22	0.941	0.356
	Female	9	52.11	9.02		
Attention (text + graph)	Male	19	52.00	11.12	0.475	0.639
	Female	9	49.89	10.68		
Attention (video)	Male	19	53.05	11.21	1.261	0.219
	Female	9	47.22	11.91		

**Table 6:** Difference comparison of attention values of learners with different education experience.

	Education	*N*	Mean	SD	*t*	*p*
Attention (text)	Undergraduate	13	51.54	13.78	-1.532	0.138
	Graduate	15	58.80	11.31		
Attention (text + graph)	Undergraduate	13	48.15	10.65	-1.472	0.153
	Graduate	15	54.07	10.57		
Attention (video)	Undergraduate	13	47.23	9.54	-1.746	0.093
	Graduate	15	54.60	12.35		

**Table 7:** Difference comparison of attention values of learners with active and reflective learning styles.

	Types	*N*	*M*	SD	*t*	*p*
Concentration (text)	Active	13	53.54	13.14	-0.720	0.478
	Reflective	15	57.07	12.75		
Concentration (text + graph)	Active	13	50.54	8.53	-0.360	0.722
	Reflective	15	52.00	12.76		
Attention (video)	Active	13	46.15	9.23	-2.308	0.029^∗^
	Reflective	15	55.53	11.86		

^∗^ *p* < 0.05.

**Table 8:** Difference comparison of attention values of learners with sequential and global learning styles.

	Types	*N*	*M*	SD	*t*	*p*
Attention (text)	Sequential	14	55.93	11.82	0.203	0.841
	Global	14	54.93	14.17		
Attention (text + graph)	Sequential	14	53.07	11.26	0.851	0.403
	Global	14	49.57	10.50		
Attention (video)	Sequential	14	52.50	13.64	0.598	0.555
	Global	14	49.86	9.36		

## Data Availability

If the original data of this study is needed, please contact the author by email (249962743@qq.com).

## References

[1] Collins A., Halverson R. (2018). *Rethinking Education in the Age of Technology: The Digital Revolution and Schooling in America*.

[2] Larasati A., Hajji A. M., Handayani A. N. (2019). Preferences analysis of engineering students on choosing learning media using support vector machine (SVM) model. *Advances in Social Science, Education and Humanities Research*.

[3] Chen M. L. (2013). *Influence factors research on mobile learning users’ continuance usage, [M.S. thesis]*.

[4] Siegenthaler E., Wurtz P., Groner R. (2010). Improving the usability of e-book readers. *Journal of Usability Studies*.

[5] Oz H. (2014). Prospective English teachers’ ownership and usage of mobile devices as m-learning tools. *Procedia - Social and Behavioral Sciences*.

[6] Sahasrabudhe V., Kanungo S. (2014). Appropriate media choice for e-learning effectiveness: role of learning domain and learning style. *Computers & Education*.

[7] Reychav I., Wu D. (2015). Mobile collaborative learning: the role of individual learning in groups through text and video content delivery in tablets. *Computers in Human Behavior*.

[8] Jonassen D. H., Peck K., Wilson B. G. (1999). *Learning with Technology: A Constructivist Approach*.

[9] Hwang G. J., Tsai C. C. (2011). Research trends in mobile and ubiquitous learning: a review of publications in selected journals from 2001 to 2010. *British Journal of Educational Technology*.

[10] Hwang G. J., Wu P. H. (2014). Applications, impacts and trends of mobile technology-enhanced learning: a review of 2008–2012 publications in selected SSCI journals. *International Journal of Mobile Learning and Organisation*.

[11] Lu T., Yang X. (2018). Effects of the visual/verbal learning style on concentration and achievement in mobile learning. *EURASIA Journal of Mathematics, Science and Technology Education*.

[12] She Y. Y., Du W. C. (2011). Research progress in educational neuroscience. *Researches in Open Education*.

[13] Solstad B. E., Aloka J., Odingo K., Alexzander D. (2007). Mobile education-a glance at the future[EB/OL]. http://www.dye.no/articles/a-glance-at-the-futere/introduction.html.

[14] Chabra T., Figueiredo J. (2002). How to design and deploy and held learning. http://www.empoweringtechnologies.net/eLearning/eLearning_exPov5_files/frame.htm.

[15] Ye C. L., Xu F. Y., Xu J. (2004). A review of mobile learning research. *E-education Research*.

[16] Parsons D., Ryu H. A framework for assessing the quality of mobile learning.

[17] Kynäslahti H. (2003). In search of elements of mobility in the context of education. *Mobile Learning*.

[18] Kukulska-Hulme A., Sharples M., Milrad M., Arnedillo-Sanchez I., Vavoula G. (2009). Innovation in mobile learning. *International Journal of Mobile and Blended Learning*.

[19] Chen C. M., Huang S. H. (2014). Web-based reading annotation system with an attention-based self-regulated learning mechanism for promoting reading performance. *British Journal of Educational Technology*.

[20] Sharples M. (2006). *Big Issues in Mobile Learning*.

[21] Chen C. M., Wang J. Y., Yu C. M. (2017). Assessing the attention levels of students by using a novel attention aware system based on brainwave signals. *British Journal of Educational Technology*.

[22] Drigas A., Ioannidou R. E., Kokkalia G., Lytras M. D. (2014). ICTs, mobile learning and social media to enhance learning for attention difficulties. *Journal of Universal Computer Science*.

[23] Lancheros-Cuesta D. J., Arias J. L. R., Forero Y. Y., Duran A. C. Evaluation of e-learning activities with NeuroSky MindWave EEG.

[24] Jamet E., Gavota M., Quaireau C. (2008). Attention guiding in multimedia learning. *Learning and Instruction*.

[25] Kinsella K. (1995). Understanding and empowering diverse learners in ESL classrooms. *Learning Styles in the ESL/EFL Classroom*.

[26] Babadoğan C. (2000). Öğretim stili odakı ders tasarımı geliştirme (in Turkish). *Milli Eğitim Dergisi*.

[27] Marković S., Jovanović N. (2012). Learning style as a factor which affects the quality of e-learning. *Artificial Intelligence Review*.

[28] Dağ F., Geçer A. (2009). Relations between online learning and learning styles. *Procedia - Social and Behavioral Sciences*.

[29] Felder R. M., Soloman B. A. (1991). *Index of Learning Styles*.

[30] Alty J. L., Al-Sharrah A., Beacham N. (2006). When humans form media and media form humans: an experimental study examining the effects different digital media have on the learning outcomes of students who have different learning styles. *Interacting with Computers*.

[31] Liu S.-H., Liao H.-L., Pratt J. A. (2009). Impact of media richness and flow on e-learning technology acceptance. *Computers & Education*.

[32] Good T. L., Brophy J. E. (1990). *Educational Psychology: A Realistic Approach*.

[33] Keskin N. O., Metcalf D. (2011). The current perspectives, theories and practices of mobile learning. *Turkish Online Journal of Educational Technology-TOJET*.

[34] Carter V. (2006). Do media influence learning? Revisiting the debate in the context of distance education. *Open Learning: The Journal of Open, Distance and e-Learning*.

[35] Hastings N. B., Tracey M. W. (2005). Does media affect learning: where are we now ?. *TechTrends*.

[36] Mayer R. E. (2002). Multimedia Learning. *Psychology of Learning and Motivation*.

[37] Clark R. E. (1994). Media will never influence learning. *Educational Technology Research and Development*.

[38] Maniar N., Bennett E., Hand S., Allan G. (2008). The effect of mobile phone screen size on video based learning. *Journal of Software*.

[39] Kozma R. B. (1994). Will media influence learning? Reframing the debate. *Educational Technology Research and Development*.

[40] Nunez P. L., Srinivasan R. (2006). A theoretical basis for standing and traveling brain waves measured with human EEG with implications for an integrated consciousness. *Clinical Neurophysiology*.

[41] Nacke L. E., Stellmach S., Lindley C. A. (2011). Electroencephalographic assessment of player experience. *Simulation & Gaming*.

[42] Wang C. C., Hsu M. C. (2014). An exploratory study using inexpensive electroencephalography (EEG) to understand flow experience in computer-based instruction. *Information & Management*.

[43] Ma M. Y., Wei C. C. (2015). A comparative study of children's concentration performance on picture books: age, gender, and media forms. *Interactive Learning Environments*.

[44] Shadiev R., Huang Y. M., Hwang J. P. (2017). Investigating the effectiveness of speech-to-text recognition applications on learning performance, attention, and meditation. *Educational Technology Research and Development*.

[45] Shadiev R., Wu T. T., Huang Y. M. (2017). Enhancing learning performance, attention, and meditation using a speech-to-text recognition application: evidence from multiple data sources. *Interactive Learning Environments*.

[46] Smithson E. F., Phillips R., Harvey D. W., Morrall M. C. H. J. (2013). The use of stimulant medication to improve neurocognitive and learning outcomes in children diagnosed with brain tumours: a systematic review. *European Journal of Cancer*.

[47] Sun J. C.-Y. (2014). Influence of polling technologies on student engagement: an analysis of student motivation, academic performance, and brainwave data. *Computers & Education*.

[48] Xu J., Zhong B. (2018). Review on portable EEG technology in educational research. *Computers in Human Behavior*.

[49] Rashida N. B. A., Taibb M. N. B., Liasb S. B. (2013). Summative EEG-based assessment of the relations between learning styles and personality traits of openness. *Procedia-Social and Behavioral Sciences*.

[50] Deenadayalan D., Kangaiammal A., Poornima B. K. EEG based learner’s learning style and preference prediction for E-learning.

[51] Rebolledo-Mendez G., Dunwell I., Martínez-Mirón E. (2009). Assessing Neurosky’s usability to detect attention levels in an assessment exercise. *International Conference on Human-Computer Interaction*.

[52] Dobosz K., Wittchen P. (2015). Brain-computer interface for mobile devices. *Journal of Medical Informatics & Technologies*.

